# Supplementary material for: Mapping evidence of food safety at transport stations in Africa: a scoping review protocol
Source: BMJ Open. 2020 Aug 11;10(8):e035879. doi: 10.1136/bmjopen-2019-035879 (PMC7418661; doi:10.1136/bmjopen-2019-035879)
Supplement: Author's manuscript [file bmjopen-2019-035879.draft_revisions.pdf]

BMJ Open is committed to open peer review. As part of this commitment we make the peer review history of every article we publish publicly available.

When an article is published we post the peer reviewers' comments and the authors' responses online. We also post the versions of the paper that were used during peer review. These are the versions that the peer review comments apply to.

The versions of the paper that follow are the versions that were submitted during the peer review process. They are not the versions of record or the final published versions. They should not be cited or distributed as the published version of this manuscript.

BMJ Open is an open access journal and the full, final, typeset and author-corrected version of record of the manuscript is available on our site with no access controls, subscription charges or pay-per-view fees (http://bmjopen.bmj.com).

If you have any questions on BMJ Open's open peer review process please email info.bmjopen@bmj.com

BMJ Open

Mapping Evidence of Food Safety at Transport stations in Africa: A Scoping Review Protocol

Journal:	*BMJ Open*
Manuscript ID	bmjopen-2019-035879
Article Type:	Protocol
Date Submitted by the Author:	20-Nov-2019

Complete List of Authors:	Ncama, Busisiwe; University of KwaZulu - Natal, School of Nursing & Public Health<br>Duma, Sinegugu; University of KwaZulu-Natal College of Health Sciences<br>Kuupiel , Desmond ; University of KwaZulu-Natal College of Health Sciences, Public Health Medicine; Research for Sustainable Development Consult,

Keywords:	Public health < INFECTIOUS DISEASES, PREVENTIVE MEDICINE, PUBLIC HEALTH

SCHOLARONE™ Manuscripts

I, the Submitting Author has the right to grant and does grant on behalf of all authors of the Work (as defined in the below author licence), an exclusive licence and/or a non-exclusive licence for contributions from authors who are: i) UK Crown employees; ii) where BMJ has agreed a CC-BY licence shall apply, and/or iii) in accordance with the terms applicable for US Federal Government officers or employees acting as part of their official duties; on a worldwide, perpetual, irrevocable, royalty-free basis to BMJ Publishing Group Ltd ("BMJ") its licensees and where the relevant Journal is co-owned by BMJ to the co-owners of the Journal, to publish the Work in this journal and any other BMJ products and to exploit all rights, as set out in our licence.

The Submitting Author accepts and understands that any supply made under these terms is made by BMJ to the Submitting Author unless you are acting as an employee on behalf of your employer or a postgraduate student of an affiliated institution which is paying any applicable article publishing charge ("APC") for Open Access articles. Where the Submitting Author wishes to make the Work available on an Open Access basis (and intends to pay the relevant APC), the terms of reuse of such Open Access shall be governed by a Creative Commons licence – details of these licences and which Creative Commons licence will apply to this Work are set out in our licence referred to above.

Other than as permitted in any relevant BMJ Author's Self Archiving Policies, I confirm this Work has not been accepted for publication elsewhere, is not being considered for publication elsewhere and does not duplicate material already published. I confirm all authors consent to publication of this Work and authorise the granting of this licence.

For peer review only

Mapping Evidence of Food Safety at Transport stations in Africa: A Scoping Review Protocol

Busisiwe P. Ncama[1], Sinegugu E. Duma[1], Desmond Kuupiel[1,2,3]

[1]College of Health Sciences, University of KwaZulu-Natal, South Africa

[2]Department of Public Health Medicine, School of Nursing and Public Health, University of KwaZulu-Natal, Durban, South Africa

[3]Research for Sustainable Development Consult, Sunyani, Ghana

Corresponding Author: Desmond Kuupiel (Ph.D.)

Address: 2nd Floor George Campbell Building, Department of Public Health Medicine, School of Nursing and Public Health, University of KwaZulu-Natal, Durban, 4001, South Africa

Email address: desmondkuupiel98@hotmail.com / KuupielD@ukzn.ac.za

Tel: +27735568200/ +233550972968

Email addresses of co-authors

Prof. Busisiwe P. Ncama (Ncamab@ukzn.ac.za)

Prof. Sinegugu E. Duma (DumaS1@ukzn.ac.za)

For peer review only

Abstract

Background: In Africa, food trading in public spaces such as transport stations, taxi ranks, and on the streets is fast-growing, particularly in the urban and peri-urban areas. Although food trading in such public spaces serves as a source of livelihood for many people, unsafe food can have a significant impact on health. We, therefore, aim to systematically map literature on food safety at transport stations in Africa and examine the literature to identify research gaps for future studies.

Methods: We will employ the Arksey & O'Malley framework, Levac et al. recommendations, and the Joanna Briggs Institute guidelines to guide this study. We will conduct a comprehensive search in: PubMed, SCOPUS, Web of Science, and Google Scholar for relevant articles using a combination of keywords/search terms with no limitations. We will also search for relevant literature from the World Health Organization and the Food and Agriculture Organization websites, and from the reference list of all included articles. Two investigators will independently screen the articles in parallel at the abstract and full-text phases using the eligibility criteria as a guide. Data extraction will be done using a piloted data extraction form designed in a Microsoft ® Word tabular format. Afterward, the extracted data will be collated into themes and sub-themes, summarized, and the results reported utilizing a narrative approach. The Preferred Reporting Items for Systematic Reviews and Meta-analysis: Extension checklist designed for scoping review will be followed to report the findings of this study.

Ethics and dissemination: Not required. All sources of data will be adequately cited and added to the reference list. We will present the final scoping review results at the appropriate workshops, meetings, conferences, as well as submit for peer review and publication in a scientific journal.

Keywords: Food safety, Food security, Transport stations, Taxi ranks, Car parks, Bus stops, Automobile stations, Africa

For peer review only - http://bmjopen.bmj.com/site/about/guidelines.xhtml

For peer review only

Article summary

Strengths and limitations of this study

- This will be the first scoping review to systematically map literature, examine the scope and identify research gaps on the safety food at transport stations in Africa
- A comprehensive search for potentially eligible articles will be conducted in four electronic databases, and websites of international organizations.
- The scoping review permits the inclusion of all types of study designs, and it encompasses both published and grey literature.
- Boolean words AND/OR as well as medical subject heading (MeSH) terms will be included in the electronic search strategy to capture all relevant studies.
- This study limitation is the use of only four databases, which may potentially result in missing relevant articles indexed in other electronic databases.

For peer review only

For peer review only - http://bmjopen.bmj.com/site/about/guidelines.xhtml

Background

Food is one of the major modes of disease transmission from microbial, chemical, and physical hazards. Unsafe food containing harmful bacteria, viruses, parasites or chemical substances can cause more than 200 different diseases – ranging from diarrhea to cancers [1]. Although unsafe food can have a significant impact on health in both high-income and low-and middle-income countries (LMICs), the impact is much higher in LMICs [2, 3]. Globally, it is estimated that unsafe food results in about 600 million cases of foodborne diseases and close to 420 000 deaths with about 30% of all foodborne deaths occurring among children less than 5 years annually [1, 2]. The WHO further estimates that eating unsafe food results in approximately 33 million years of healthy lives lost (DALYs) worldwide every year [1]. As a result, in 2006, the World Health Organization (WHO) declared foodborne diseases as well as food-related diseases and food safety as a major public health concern [4, 5]. Unsafe food creates a vicious cycle of disease and malnutrition, particularly affecting infants, young children, the elderly and the sick. To this end, access to safe, and nutritious and sufficient food is key to sustaining life and promoting good health.

Food safety according to the WHO denotes restraining the presence of hazards whether chronic or acute, that may make food harmful to the health of the consumer [6]. Food safety is about producing, handling, storing and preparing food in such a way as to prevent infection and contamination in the food production chain, and to help ensure that food quality and wholesomeness are maintained to promote good health [6]. In addition to contributing to food and nutrition security, a safe food supply also supports national economies, trade, and tourism, stimulating sustainable development [6, 7]. The globalization of food trade, a growing world population, climate change and rapidly changing food systems have an impact on the safety of food [6, 7].

In most LMICs, food trading in public spaces such as transport stations, taxi ranks, and on the streets is fast-growing, particularly in the urban and peri-urban areas. It is evident that over 2.4 billion people eat food sold by vendors in those public spaces every day worldwide [8, 9] due to several reasons such as the thinking that the foods sold in public spaces are appealing, ready-to-eat, convenience, and of low cost and savory taste [10-12]. Food trading in public spaces serves as a source of livelihood for many households in LMICs [7, 10, 12]. Although the food sold in public spaces may also have great nutritional value depending on the type of ingredients and the conditions under which the food was prepared and served to the customer, there are health implications. This is mostly because the food is prepared and sold in an environment

For peer review only - http://bmjopen.bmj.com/site/about/guidelines.xhtml

that often lacks sanitary facilities and surveillance, and at times, has no legal control in most LMICs [12-14]. Despite this, no study to the best of our knowledge has map literature on food safety in public spaces in Africa. We, therefore, intend to conduct a systematic search for literature to examine the scope of evidence and identify research gaps on food safety in public spaces with a focus on transport stations in Africa. We expect to retrieve many studies and describe their findings to inform policy. We also anticipate identifying gaps for future research to improve food safety in public spaces such as taxi ranks, transport stations, and the streets of Africa.

Methods

Design of the study protocol

We will employ an amended Arksey & O'Malley framework incorporating the Levac et. al. recommendation [15, 16] to guide this scoping review. The Arksey & O'Malley framework includes the following: research question identification; identifying relevant studies; selection of study; data charting, collating, summarising and reporting the findings, and consultation. However, we will exclude the consultation stage because this review is nested in a larger study that already includes stakeholder consultation.

Identifying the research question

This scoping review question shall be:

What is the scope of evidence on food safety and public spaces in Africa? Table 1 presents the Population, Concept, and Context (PCC) mnemonic used to inform the eligibility of this proposed review [17].

Identify relevant studies

We will conduct a comprehensive search in: PubMed, SCOPUS, Web of Science, and Google Scholar for relevant articles using a combination of the following keywords: ("transport stations", "taxi ranks", "automobile stations" and "streets" "public space", "food safety", "food handling", "food storage", "food preparation", "vending" "selling","food trading", "hawking", "health", "safety", and "Africa"). We will also search by using individual countries' names in Africa in order to enable us to retrieve all appropriate articles. Additional search terms/keywords may be identified and incorporated into the search strategy in consultation with a Librarian. We will include booleans terms AND/OR and medical subject heading (MeSH)

For peer review only - http://bmjopen.bmj.com/site/about/guidelines.xhtml

terms in our search. Date, study design, and language limitations will be removed during the databases search. The websites of International Organisations such as the WHO, and the Food and Agriculture Organisation will also be searched for relevant articles. We will further search for relevant grey literature such as unpublished manuscripts, thesis, and dissertations using Google. Moreover, we will search the reference list of all included studies for articles presenting useful information to address this propose scoping review question. The search date, database, keywords, number of retrieved articles, number of eligible articles, and all other relevant search records will be documented appropriately in detail. Table 2 illustrates a pilot search in PubMed database for this proposed scoping review study.

Eligibility criteria and study selection

The eligibility criteria will be as outlined below.

Inclusion criteria:

This shall include the following:

- Articles reporting evidence from Africa,
- Articles presenting evidence on this study's population,
- Articles presenting evidence on food safety or security,
- Articles reporting evidence on health-related issues (Example, foodborne diseases)
- Primary studies

Exclusion criteria

We shall exclude the following:

- Articles presenting evidence from other countries outside Africa,
- Articles presenting evidence on food trading in homes, restaurants, markets, hotels, and others not included in this study population,
- Articles presenting evidence on agriculture and climate change,
- Articles presenting evidence on non-health topics
- Review studies

Study selection

The screening for potentially eligible studies will be done in three phases. In phase one, a comprehensive articles search in the electronic databases will be conducted by one investigator and import all eligibility studies to a new endnotes X9 library created for the review. To clean

For peer review only - http://bmjopen.bmj.com/site/about/guidelines.xhtml

the library, all duplicates will be deleted and subsequently shared with the co-investigators. In phase two, two investigators will independently screen the abstracts in parallel guided by the eligibility criteria designed using a google form. All disagreements between the investigators in terms of their response at this stage will be addressed through discussions among the investigators until a consensus is reached. Similarly, in the third phase, two investigators will independently screen all the full-text articles as described in phase two, however, a third investigator will be engaged to resolve any discrepancies in their responses. We shall collaborate with the systematic review unit of the University of KwaZulu-Natal to articles published in other languages either English. All articles retrieved from the reference list of included studies will also be screened using the same process. Where a full-text article cannot be retrieved or not accessible from the online databases, we will seek assistance from the University of KwaZulu-Natal library or write to the authors to request the full-text for screening. An amended PRISMA (Preferred Reporting Items for Systematic Review and Meta-Analyses) flow diagram will be used to report the search and screening results [18] (Figure 1).

Charting the data

We will design a data extraction form using Microsoft ® Word in a tabular format. To ensure the trustworthiness and reliability of the data, two independent investigators will pilot the data extraction form using a random sample of ten percent of the included articles in parallel. Subsequently, the data extraction form will be revised if necessary to ensure its rigor and ability to capture all relevant data to answer the review question. We will keep the data extraction form updated throughout this process until all relevant information has been extracted from the included articles. Table 3 shows the data extraction form that will be used for this scoping review study.

Collating, summarizing, and reporting the results

We will present information about the included articles that are aligned with the research question of this proposed scoping review study. All relevant data from the eligible studies will be extracted manually using the piloted data-extraction form to answer the review question. Following this process, the extracted data will be collated into themes and sub-themes, summarise, and the results presented using a narrative approach. The PRISMA extension designed for the scoping reviews checklist will be utilized to guide the reporting of the results of this scoping review. Afterward, a meta-analysis may be conducted using quantitative data if possible.

For peer review only - http://bmjopen.bmj.com/site/about/guidelines.xhtml

Conclusion

This manuscript presents the protocol of a scoping review study. We anticipate the scoping review results will provide useful evidence-based information to inform for food safety policy decisions and implementation on food trading in transport stations, taxi ranks, and the streets in Africa. This scoping review will also permit us to examine the available evidence and identify gaps in the literature and make recommendations for future research such as systematic reviews, meta-analysis, and primary studies in Africa.

Ethics and dissemination

Not required. All sources of data will be adequately referenced.

Statements

Acknowledgments

We would also like to thank the University of KwaZulu-Natal for training and for the resources used in the development of this protocol.

Authors' contributions

BPN, SED, and DK conceptualized the study. DK wrote the manuscript. BPN and SED contributed to the writing. All authors critically reviewed and approved the final manuscript.

Funding

This research received no specific grant from any funding agency in the public, commercial or not-for-profit sectors

Competing interests

None declared

References

1. World Health Organization. Estimating the burden of foodborne diseases Geneva: World Health Organization; 2015 [cited 2019 25/10/2019]. Available from: https://www.who.int/activities/estimating-the-burden-of-foodborne-diseases.

For peer review only - http://bmjopen.bmj.com/site/about/guidelines.xhtml

2. The Lancet Gastroenterology H. Food safety really is everyone's business. The Lancet Gastroenterology & hepatology. 2019;4(8):571.

3. Allard DG. The 'farm to plate' approach to food safety - Everyone's business. The Canadian journal of infectious diseases = Journal canadien des maladies infectieuses. 2002;13(3):185-90.

4. World Health Organization. Five keys to safer food manual Geneva: World Health Organization; 2006 [cited 2019 25/10/2019]. Available from: https://www.who.int/foodsafety/publications/consumer/manual_keys.pdf.

5. Lamin-Boima P. Knowledge, Attitude And Practice Of Street Food Vendors In Selected Schools Within Bo City Southern Sierra Leone.

6. Organization WH. Food Safety: What you should know Geneva: World Health Organization; 2015 [cited 2019 25/10/2019]. Available from: http://www.searo.who.int/entity/world_health_day/2015/whd-what-you-should-know/en/.

7. Hill J, McHiza Z, Puoane T, Steyn NP. The development of an evidence-based street food vending model within a socioecological framework: A guide for African countries. PloS one. 2019;14(10):e0223535.

8. Food and Agriculture Organization. Selling street and snack foods Rome: Food and Agriculture Organization; 2011 [cited 2019 25/10/2019]. Available from: http://www.fao.org/3/a-i2474e.pdf.

9. Gelormini M, Damasceno A, Lopes SA, Malo S, Chongole C, Muholove P, et al. Street Food Environment in Maputo (STOOD Map): a Cross-Sectional Study in Mozambique. JMIR research protocols. 2015;4(3):e98.

10. Liu Z, Zhang G, Zhang X. Urban street foods in Shijiazhuang city, China: Current status, safety practices and risk mitigating strategies. Food Control. 2014;41:212-8.

11. Al Mamun M, Turin TC. Safety of Street foods. Food Hygiene and Toxicology in Ready-to-Eat Foods: Elsevier; 2016. p. 15-29.

12. Abrahale K, Sousa S, Albuquerque G, Padrao P, Lunet N. Street food research worldwide: a scoping review. Journal of human nutrition and dietetics: the official journal of the British Dietetic Association. 2019;32(2):152-74.

For peer review only - http://bmjopen.bmj.com/site/about/guidelines.xhtml

13. Ekanem EO. The street food trade in Africa: safety and socio-environmental issues. Food Control. 1998;9(4):211-5.

14. Rane S. Street vended food in developing world: hazard analyses. Indian journal of microbiology. 2011;51(1):100-6.

15. Arksey H, O'Malley L. Scoping studies: towards a methodological framework. International Journal of Social Research Methodology. 2005;8(1):19-32.

16. Levac D, Colquhoun H, O'Brien KK. Scoping studies: advancing the methodology. Implementation Science. 2010;5(1):69.

17. Institute JB. Joanna Briggs Institute reviewers' manual: 2015 edition/supplement. Methodology for JBI Scoping Reviews Adelaide: The Joanna Briggs Institute. 2015.

18. Moher D, Liberati A, Tetzlaff J, Altman DG, The PRISMA Group. PRISMA 2009 Flow Diagram. 2009;6(2009):1000097.

Tables

Table 1: PCC framework for defining the eligibility of the studies for the primary research question

P- Population	Transport stations: This will include bus stations, lorry parks, taxi ranks, and automobile stations, and Bus stops.
C-Concept	Food safety: This will be defined in terms of food handling, storing and preparing food, and food vending or hawking in such a way as to prevent infection and contamination in the food production chain, and to help ensure that food quality and wholesomeness are maintained to promote good health.
C-Context	African

For peer review only - http://bmjopen.bmj.com/site/about/guidelines.xhtml

Table 2: Pilot search in PubMed electronic database

Date	Database	Keywords	Search results
28/10/2019	PubMed	("food safety"[MeSH Terms] OR ("food"[All Fields] AND "safety"[All Fields]) OR "food safety"[All Fields]) OR ("food supply"[MeSH Terms] OR ("food"[All Fields] AND "supply"[All Fields]) OR "food supply"[All Fields] OR ("food"[All Fields] AND "security"[All Fields]) OR "food security"[All Fields]) AND (("food"[MeSH Terms] OR "food"[All Fields]) AND vending[All Fields]) OR (("food"[MeSH Terms] OR "food"[All Fields]) AND trading[All Fields]) AND (("motor vehicles"[MeSH Terms] OR ("motor"[All Fields] AND "vehicles"[All Fields]) OR "motor vehicles"[All Fields] OR "lorry"[All Fields]) AND parks[All Fields]) OR (("motor vehicles"[MeSH Terms] OR ("motor"[All Fields] AND "vehicles"[All Fields]) OR "motor vehicles"[All Fields] OR "lorry"[All Fields]) AND station[All Fields]) OR (taxi[All Fields] AND ranks[All Fields])	261

For peer review only - http://bmjopen.bmj.com/site/about/guidelines.xhtml

Table 3: Data Extraction Form

Author and publication year
Study Title
Study aim/objective
Type of study design
Study setting (Country)
Place of food trading
Study participants
Significant findings
Food safety/security outcomes
Conclusions/recommendations

For peer review only - http://bmjopen.bmj.com/site/about/guidelines.xhtml

Figure

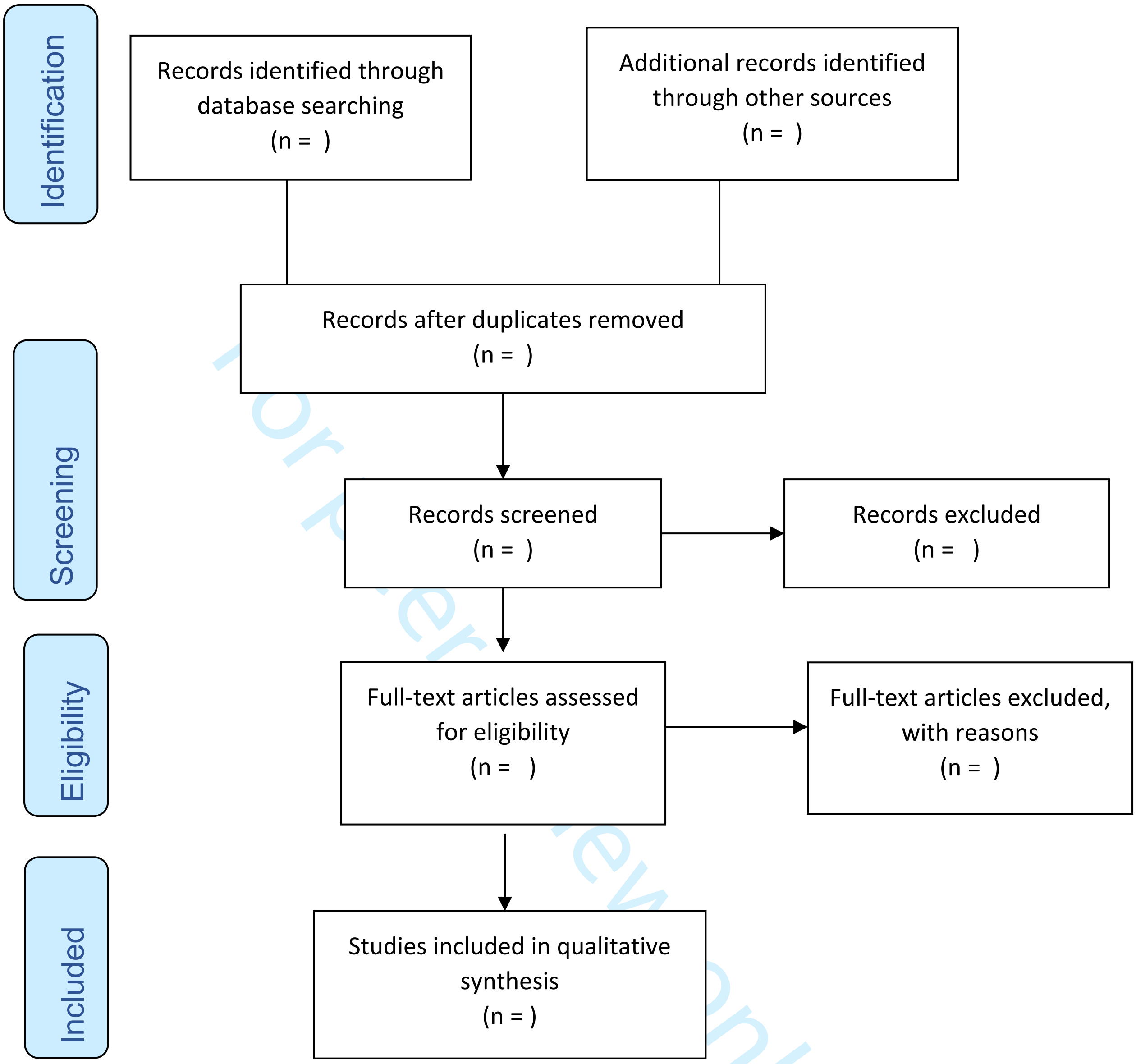

Figure 1: PRISMA 2009 Flow Diagram [18]

For peer review only - http://bmjopen.bmj.com/site/about/guidelines.xhtml

Mapping Evidence of Food Safety at Transport stations in Africa: A Scoping Review Protocol

Journal:	*BMJ Open*
Manuscript ID	bmjopen-2019-035879.R1
Article Type:	Protocol
Date Submitted by the Author:	15-Apr-2020
Complete List of Authors:	Ncama, Busisiwe; University of KwaZulu-Natal College of Health Sciences<br>Kuupiel , Desmond ; University of KwaZulu-Natal College of Health Sciences, Public Health Medicine; Research for Sustainable Development Consult,<br>Duma, Sinegugu; University of KwaZulu-Natal College of Health Sciences<br>Mchunu , Gugu ; University of KwaZulu-Natal College of Health Sciences<br>Guga , Phindile ; University of KwaZulu-Natal College of Health Sciences<br>Slotow, Rob; University of KwaZulu-Natal
**Primary Subject Heading**:	Public health
Secondary Subject Heading:	Health policy, Public health, Evidence based practice
Keywords:	Public health < INFECTIOUS DISEASES, PREVENTIVE MEDICINE, PUBLIC HEALTH, Health policy < HEALTH SERVICES ADMINISTRATION & MANAGEMENT, Health & safety < HEALTH SERVICES ADMINISTRATION & MANAGEMENT

SCHOLARONE™
Manuscripts

I, the Submitting Author has the right to grant and does grant on behalf of all authors of the Work (as defined in the below author licence), an exclusive licence and/or a non-exclusive licence for contributions from authors who are: i) UK Crown employees; ii) where BMJ has agreed a CC-BY licence shall apply, and/or iii) in accordance with the terms applicable for US Federal Government officers or employees acting as part of their official duties; on a worldwide, perpetual, irrevocable, royalty-free basis to BMJ Publishing Group Ltd ("BMJ") its licensees and where the relevant Journal is co-owned by BMJ to the co-owners of the Journal, to publish the Work in this journal and any other BMJ products and to exploit all rights, as set out in our licence.

The Submitting Author accepts and understands that any supply made under these terms is made by BMJ to the Submitting Author unless you are acting as an employee on behalf of your employer or a postgraduate student of an affiliated institution which is paying any applicable article publishing charge ("APC") for Open Access articles. Where the Submitting Author wishes to make the Work available on an Open Access basis (and intends to pay the relevant APC), the terms of reuse of such Open Access shall be governed by a Creative Commons licence – details of these licences and which Creative Commons licence will apply to this Work are set out in our licence referred to above.

Other than as permitted in any relevant BMJ Author's Self Archiving Policies, I confirm this Work has not been accepted for publication elsewhere, is not being considered for publication elsewhere and does not duplicate material already published. I confirm all authors consent to publication of this Work and authorise the granting of this licence.

For peer review only

Mapping Evidence of Food Safety at Transport stations in Africa: A Scoping Review Protocol

Busisiwe P. Ncama[1], Desmond Kuupiel[1,2], Sinegugu E. Duma[1], Gugu Mchunu[1], Phindile Guga[1], Rob Slotow[3]

[1]School of Nursing and Public Health, College of Health Sciences, University of KwaZulu-Natal, Durban, South Africa

[2]Research for Sustainable Development Consult, Sunyani, Ghana

[3]School of Life Scienes, College of Agriculture, Engeering and Science, Universitity of Kwazulu-Natal, Pietermaritzburg, South Africa.

Corresponding Author: Desmond Kuupiel (Ph.D.)

Address: 2nd Floor George Campbell Building, Department of Public Health Medicine, School of Nursing and Public Health, University of KwaZulu-Natal, Durban, 4001, South Africa

Email address: desmondkuupiel98@hotmail.com / KuupielD@ukzn.ac.za

Tel: +27735568200/ +233550972968

Email addresses of co-authors

Prof. Busisiwe P. Ncama (Ncamab@ukzn.ac.za)

Prof. Sinegugu E. Duma (DumaS1@ukzn.ac.za)

Dr. Gugu Mchunu (Mchunug@ukzn.ac.za)

Ms. Phindile Guga (phindile.guga@gmail.com)

Prof. Rob Slotow (Slotow@ukzn.ac.za)

For peer review only

For peer review only - http://bmjopen.bmj.com/site/about/guidelines.xhtml

Abstract

Introduction: In Africa, travels, urbanization, and changing consumer habits, are increasing the number of people buying and eating food prepared/sold at public spaces including transport stations, particularly in the urban and peri-urban areas. Although food trading in such public spaces serves as a source of livelihood for many people, unsafe food can have a negative impact on health. We, therefore, aim to systematically explore and examine the literature, and describe the evidence on food safety (food handling, storage, preparation and sale, packaging of food when sold, hygiene of sale venue, and quality (nutrition) of food sold/purchased/eaten) at transport stations to inform policy, as well as identify research gaps for future studies in Africa.

Methods and Analysis: We will employ the Arksey & O'Malley framework, Levac et al. recommendations, and the Joanna Briggs Institute guidelines to guide this study. We will conduct a comprehensive search in PubMed, SCOPUS, Web of Science, Google Scholar, and EBSCOhost (Academic search complete, CINAHL with Full-text, and Health Source) from inception to December 2019 for relevant peer-review articles using a combination of keywords/search terms with no limitations. We will also search for relevant literature from the reference list of all included articles. Two investigators will independently screen the articles in parallel at the abstract and full-text phases using the eligibility criteria as a guide. Data extraction will be done using a piloted data extraction form designed in a Microsoft ® Word tabular format. Afterward, the extracted data will be collated into themes and sub-themes, summarized, and the results reported utilizing a narrative approach. We will the Preferred Reporting Items for Systematic Reviews and Meta-analyses: Extension for scoping reviews checklist to report this study results.

Ethics and Dissemination: Ethics approval not required. All sources of data will be adequately cited and added to the reference list. We will present the final scoping review results at the appropriate workshops, meetings, conferences, as well as submit for peer review and publication in a scientific journal.

Keywords: Food safety, Transport stations, Taxi ranks, Car parks, Bus stops, Automobile stations, Africa

For peer review only - http://bmjopen.bmj.com/site/about/guidelines.xhtml

Article summary

Strengths and limitations of this study

- This will be the first scoping review to systematically explore literature and describe the evidence on food safety at transport stations to inform policy, as well as identify research gaps for future studies in Africa
- A comprehensive search for potentially eligible articles will be conducted in four electronic databases, and websites of international organizations.
- The scoping review permits the inclusion of all types of study designs, and it encompasses both published and grey literature.
- Boolean words AND/OR as well as medical subject heading (MeSH) terms will be included in the electronic search strategy to capture all relevant studies.
- This study limitation is the use of only four databases, which may potentially result in missing relevant articles indexed in other electronic databases.

For peer review only - http://bmjopen.bmj.com/site/about/guidelines.xhtml

Background

Food is one of the major modes of disease transmission for microbial, chemical, and physical hazards. Food containing harmful bacteria, viruses, parasites or chemical substances can cause more than 200 different diseases – ranging from diarrhea to cancers [1]. Although unsafe food can have a significant impact on health in both high-income and low-and middle-income countries (LMICs), the impact is much higher in LMICs [2, 3]. Globally, it is estimated that unsafe food results in about 600 million cases of foodborne diseases and close to 420 000 deaths with about 30% of all foodborne deaths occurring among children less than 5 years annually [1, 2]. The WHO further estimates that eating unsafe food results in approximately 33 million years of healthy lives lost (DALYs) worldwide every year [1]. As a result, in 2006, the World Health Organization (WHO) declared foodborne diseases as well as food-related diseases and food safety as a major public health concern [4, 5]. Unsafe food creates a vicious cycle of disease and malnutrition, particularly affecting infants, young children, the elderly and the sick [6]. To this end, access to safe, and nutritious and sufficient food is key to sustaining life and promoting good health.

Food safety may denote the prevention of biological or non-biological hazards in food , whether chronic or acute, that may make food harmful to the health of the consumer [7]. Food safety is about producing, handling, storing and preparing food in such a way as to prevent infection and contamination in the food production chain, and to help ensure that food quality and wholesomeness are maintained to promote good health [7]. In addition to contributing to food and nutrition security, a safe food supply also supports national economies, trade, and tourism, stimulating sustainable development [7, 8]. The globalization of food trade, a growing world population, climate change and rapidly changing food systems have an impact on the safety of food [7, 8].

In most LMICs, food trading at transport stations such as taxi ranks, bus stations, and lorry parks is fast-growing, particularly in the urban and peri-urban areas. It is evident that over 2.4 billion people eat food sold by vendors at public spaces including transport stations every day worldwide [9, 10]. This may be due to several reasons such as the thinking that the foods sold in public spaces are appealing, ready-to-eat, convenience, and of low cost and savory taste [11-13]. Food trading in public spaces serves as a source of livelihood for many households in LMICs [8, 11, 13]. Although the food sold at transport stations may also have great nutritional value depending on the type of ingredients and the conditions under which the food was prepared and served to the customer, it may as well cause ill-health arising from contamination

For peer review only - http://bmjopen.bmj.com/site/about/guidelines.xhtml

of the food. Since the food is most often prepared and sold in an environment that often lacks sanitary facilities and health surveillance, and at times, not regulated by the appropriate local authorities in most LMICs [13-15]. Despite this, no study to the best of our knowledge has mapped literature on food safety at transport stations in Africa. We, therefore, intend to systematically search for literature, examine the scope of the literature and describe the evidence on food safety at transport stations in Africa in order to inform policy. We also anticipate identifying gaps from the study findings for future research to improve food safety at transport stations such as taxi ranks, bus stations, and lorry parks in Africa.

Methods

Design of the study protocol

We will employ an amended Arksey & O'Malley framework incorporating the Levac et. al. recommendation [16, 17] to guide this scoping review. The Arksey & O'Malley framework includes the following: research question identification; identifying relevant studies; selection of study; data charting, collating, summarising and reporting the findings, and consultation. However, we will exclude the consultation stage because this review is nested in a larger study that already includes stakeholder consultation. We followed the preferred reporting items for systematic and meta-analysis extension for protocols (PRISMA-P) guidelines to developed this protocol (Supplementary file 1). However, we will the Preferred Reporting Items for Systematic Reviews and Meta-analyses: Extension for scoping reviews checklist to report this study results.

Identifying the research question

This scoping review question shall be:

What is the scope of evidence on food safety at transport stations in Africa? Table 1 presents the Population, Concept, and Context (PCC) mnemonic used to inform the eligibility of this proposed review [18].

Identify relevant studies

We will conduct a comprehensive search in: PubMed, SCOPUS, Google scholar, Web of Science, and EBSCOhost (Academic search complete, CINAHL with Full-text, and Health Source) from inception to December 2019 for relevant articles. We will develop the search strategy in consultation with a subject Librarian at the University of KwaZulu-Natal so as to enable us capture all/most relevant articles for the study. We will use a combination of the

For peer review only - http://bmjopen.bmj.com/site/about/guidelines.xhtml

following keywords: “transport station” OR “taxi rank” OR “automobile station” OR “bus station” OR “lorry park” OR “car park” OR “transport hub” OR “streets” OR “public space” AND "food safety" OR "food handling" OR "food storage" OR “food preparation” OR “food packaging” OR “food sale” OR “food vend” OR “food sale” OR “food sold” OR “food hawking” OR “food purchase” in order to enable us capture all appropriate peer-review articles. We will include booleans terms AND/OR and medical subject heading (MeSH) terms in our search. Date, study design, and language limitations will be removed during the databases search. We will also search the reference list of all included studies for articles presenting useful information to address this proposed scoping review question. The search date, database, keywords, number of retrieved articles, number of eligible articles, and all other relevant search records will be documented appropriately in detail. Table 2 illustrates a full search strategy in PubMed database for this proposed scoping review study.

Eligibility criteria and study selection

The eligibility criteria will be as outlined below.

Inclusion criteria:

This shall include the following:

- Studies reporting evidence from Africa,
- Studies presenting evidence on food safety,
- Studies reporting from transport stations,
- Studies published in English, and
- Primary studies focusing on food safety at transportations

Exclusion criteria

We shall exclude the following:

- Studies presenting evidence from other countries outside Africa,
- Studies presenting evidence of food safety from other sites such as homes, restaurants, markets, and hotels, and schools,
- Studies published in other languages such as French, Arabic, and Portuguese , and
- Review studies

Study selection

The screening for potentially eligible studies will be done in three phases. In phase one, a

For peer review only - http://bmjopen.bmj.com/site/about/guidelines.xhtml

187 comprehensive articles search in the electronic databases will be conducted by one investigator
188 and import all eligible articles to a new endnotes X9 library created for the review. To clean
189 the library, all duplicates will be deleted and subsequently shared with the co-investigators. In
190 phase two, two investigators will independently screen the abstracts in parallel guided by the
191 eligibility criteria designed using a google form. All disagreements between the investigators
192 in terms of their response at this stage will be addressed through discussions among the
193 investigators until a consensus is reached. Similarly, in the third phase, two investigators will
194 independently screen all the full-text articles as described in phase two, however, a third
195 investigator will be engaged to resolve any discrepancies in their responses. Cohen's kappa
196 statistic will be calculated following full text screening to demonstrate the level of agreement
197 between reviewers. All articles retrieved from the reference list of included studies will also be
198 screened using the same process. Where a full-text article cannot be retrieved or not accessible
199 from the online databases, we will seek assistance from the University of KwaZulu-Natal
200 library or write to the authors to request the full-text for screening. An amended PRISMA
201 (Preferred Reporting Items for Systematic Review and Meta-Analyses) flow diagram will be
202 used to report the search and screening results [19] (Figure 1).

203 **Charting the data**

204 We will design a data extraction form using Microsoft ® Word in a tabular format. To ensure
205 the trustworthiness and reliability of the data, two independent investigators will pilot the data
206 extraction form using a random sample of ten percent of the included articles in parallel.
207 Subsequently, the data extraction form will be revised if necessary to ensure its rigor and ability
208 to capture all relevant data to answer the review question. We will keep the data extraction
209 form updated throughout this process until all relevant information has been extracted from the
210 included articles. Table 3 shows the data extraction form that will be used for this scoping
211 review study.

213 **Collating, summarizing, and reporting the results**

214 We will present information about the included articles that are aligned with the research
215 question of this proposed scoping review study. All relevant data from the eligible studies will
216 be extracted using the piloted data-extraction form to answer the review question. Both
217 deductive (for characteristic of the included articles) and inductive (for study
218 findings/outcomes) approaches will be used to extract relevant data. Thematic analysis will be

For peer review only - http://bmjopen.bmj.com/site/about/guidelines.xhtml

utilised to collated the study findings into themes and sub-themes. Then, a narrative summary of the themes and sub-themes will be reported. To ensure rigor, the analysis will be conducted by two investigators with input from collaborators. The PRISMA extension designed for the scoping reviews checklist will be utilized to guide the reporting of the results of this scoping review. Afterward, a meta-analysis may be conducted using quantitative data if possible.

Conclusion

This manuscript presents the protocol of a scoping review study. This scoping review will permit us to systematically search for literature, examine the scope of the literature and describe the available evidence on food safety at transport stations in Africa. We anticipate the scoping review results will provide useful evidence-based information to inform food safety policy decisions and implementation at transport stations in Africa. This study will also permit us to identify gaps in the literature and make recommendations for future research on food safety at transport stations in Africa for further inform policy.

Ethics and dissemination

Not required. All sources of data will be adequately referenced. We will present the final scoping review results at the appropriate workshops, meetings, conferences, as well as submit for peer review and publication in a scientific journal.

Patient and Public Involvement

No patient involved

Statements

Acknowledgments

Support for this work was provided by the Sustainable and Healthy Food Systems (SHEFS) Programme, supported through the Wellcome Trust's Our Planet, Our Health Programme

Authors' contributions

BPN, DK, SED, SM, and RS conceptualized the study. DK wrote the manuscript and PG contributed to the writing. BPN, SED, GM, and RS critically review the manuscript and made revisions. DK wrote the final draft manuscript and all the authors approved it.

For peer review only - http://bmjopen.bmj.com/site/about/guidelines.xhtml

Funding

Funding for this work was provided by the Sustainable and Healthy Food Systems (SHEFS) Programme, supported through the Wellcome Trust's Our Planet, Our Health Programme [grant number: 205200/Z/16/Z]. The funder played no role in the literature search and writing of the manuscript.

Competing interests

None declared

References

1. World Health Organization. Estimating the burden of foodborne diseases Geneva: World Health Organization; 2015 [cited 2019 25/10/2019]. Available from: https://www.who.int/activities/estimating-the-burden-of-foodborne-diseases.

2. The Lancet Gastroenterology H. Food safety really is everyone's business. The lancet Gastroenterology & hepatology. 2019;4(8):571.

3. Allard DG. The 'farm to plate' approach to food safety - Everyone's business. The Canadian journal of infectious diseases = Journal canadien des maladies infectieuses. 2002;13(3):185-90.

4. World Health Organization. Five keys to safer food manual Geneva: World Health Organization; 2006 [cited 2019 25/10/2019]. Available from: https://www.who.int/foodsafety/publications/consumer/manual_keys.pdf.

5. Lamin-Boima P. Knowledge, Attitude And Practice Of Street Food Vendors In Selected Schools Within Bo City Southern Sierra Leone.

6. World Health Organization. Food safety Geneva: World Health Organization; 2019 [cited 2020 25/03/2020]. Available from: https://www.who.int/news-room/fact-sheets/detail/food-safety.

7. Organization WH. Food Safety: What you should know Geneva: World Health Organization; 2015 [cited 2019 25/10/2019]. Available from: http://www.searo.who.int/entity/world_health_day/2015/whd-what-you-should-know/en/.

For peer review only - http://bmjopen.bmj.com/site/about/guidelines.xhtml

8. Hill J, McHiza Z, Puoane T, Steyn NP. The development of an evidence-based street food vending model within a socioecological framework: A guide for African countries. PloS one. 2019;14(10):e0223535.

9. Food and Agriculture Organization. Selling street and snack foods Rome: Food and Agriculture Organization; 2011 [cited 2019 25/10/2019]. Available from: http://www.fao.org/3/a-i2474e.pdf.

10. Gelormini M, Damasceno A, Lopes SA, Malo S, Chongole C, Muholove P, et al. Street Food Environment in Maputo (STOOD Map): a Cross-Sectional Study in Mozambique. JMIR research protocols. 2015;4(3):e98.

11. Liu Z, Zhang G, Zhang X. Urban street foods in Shijiazhuang city, China: Current status, safety practices and risk mitigating strategies. Food Control. 2014;41:212-8.

12. Al Mamun M, Turin TC. Safety of Street foods. Food Hygiene and Toxicology in Ready-to-Eat Foods: Elsevier; 2016. p. 15-29.

13. Abrahale K, Sousa S, Albuquerque G, Padrao P, Lunet N. Street food research worldwide: a scoping review. Journal of human nutrition and dietetics : the official journal of the British Dietetic Association. 2019;32(2):152-74.

14. Ekanem EO. The street food trade in Africa: safety and socio-environmental issues. Food Control. 1998;9(4):211-5.

15. Rane S. Street vended food in developing world: hazard analyses. Indian journal of microbiology. 2011;51(1):100-6.

16. Arksey H, O'Malley L. Scoping studies: towards a methodological framework. International Journal of Social Research Methodology. 2005;8(1):19-32.

17. Levac D, Colquhoun H, O'Brien KK. Scoping studies: advancing the methodology. Implementation Science. 2010;5(1):69.

18. Institute JB. Joanna Briggs Institute reviewers' manual: 2015 edition/supplement. Methodology for JBI Scoping Reviews Adelaide: The Joanna Briggs Institute. 2015.

19. Moher D, Liberati A, Tetzlaff J, Altman DG, The PRISMA Group. PRISMA 2009 Flow Diagram. 2009;6(2009):1000097.

For peer review only - http://bmjopen.bmj.com/site/about/guidelines.xhtml

Tables

Table 1: PCC framework for defining the eligibility of the studies for the primary research question

P- Population	Transport stations: This will include bus stations, lorry parks, taxi ranks, and automobile stations, and bus stops.
C-Concept	Food safety: This include food handling, storage, preparation and sale, packaging of food when sold, hygiene of sale venue, and quality (nutrition) of food sold/purchased/eaten.
C-Context	African

For peer review only - http://bmjopen.bmj.com/site/about/guidelines.xhtml

Table 2: A full search strategy in PubMed illustrating the feasibility of the proposed scoping review

**Date**	**Database**	**Keywords**	**Search results**
06/04/2020	PubMed	(("transport"[All Fields]) AND station[All Fields]) OR (taxi[All Fields] AND rank[All Fields]) OR (("automobiles"[MeSH Terms] OR "automobiles"[All Fields] OR "automobile"[All Fields]) AND station[All Fields]) OR "bus station"[All Fields] OR (("motor vehicles"[MeSH Terms] OR ("motor"[All Fields] AND "vehicles"[All Fields]) OR "motor vehicles"[All Fields] OR "lorry"[All Fields]) AND park[All Fields]) OR "car park"[All Fields] OR "transport hub"[All Fields] OR "streets"[All Fields] OR "public space"[All Fields] AND "food safety"[All Fields] OR "food handling"[All Fields] OR "food storage"[All Fields] OR "food preparation"[All Fields] OR "food packaging"[All Fields] OR "food sale"[All Fields] OR (("food"[MeSH Terms] OR "food"[All Fields]) AND vend[All Fields]) OR "food sale"[All Fields] OR "food sold"[All Fields] OR (("food"[MeSH Terms] OR "food"[All Fields]) AND ("hawks"[MeSH Terms] OR "hawks"[All Fields] OR "hawking"[All Fields])) OR "food purchase"[All Fields] AND (("0000/01/01"[PDAT] : "2019/12/31"[PDAT]) AND "humans"[MeSH Terms])	13644

For peer review only

Table 3: Data Extraction Form

Author and publication year
Study Title
Study aim/objective
Type of study design
Study setting (Country)
Study participants
Significant findings
Food safety outcomes
Conclusions/recommendations

For peer review only

Figure

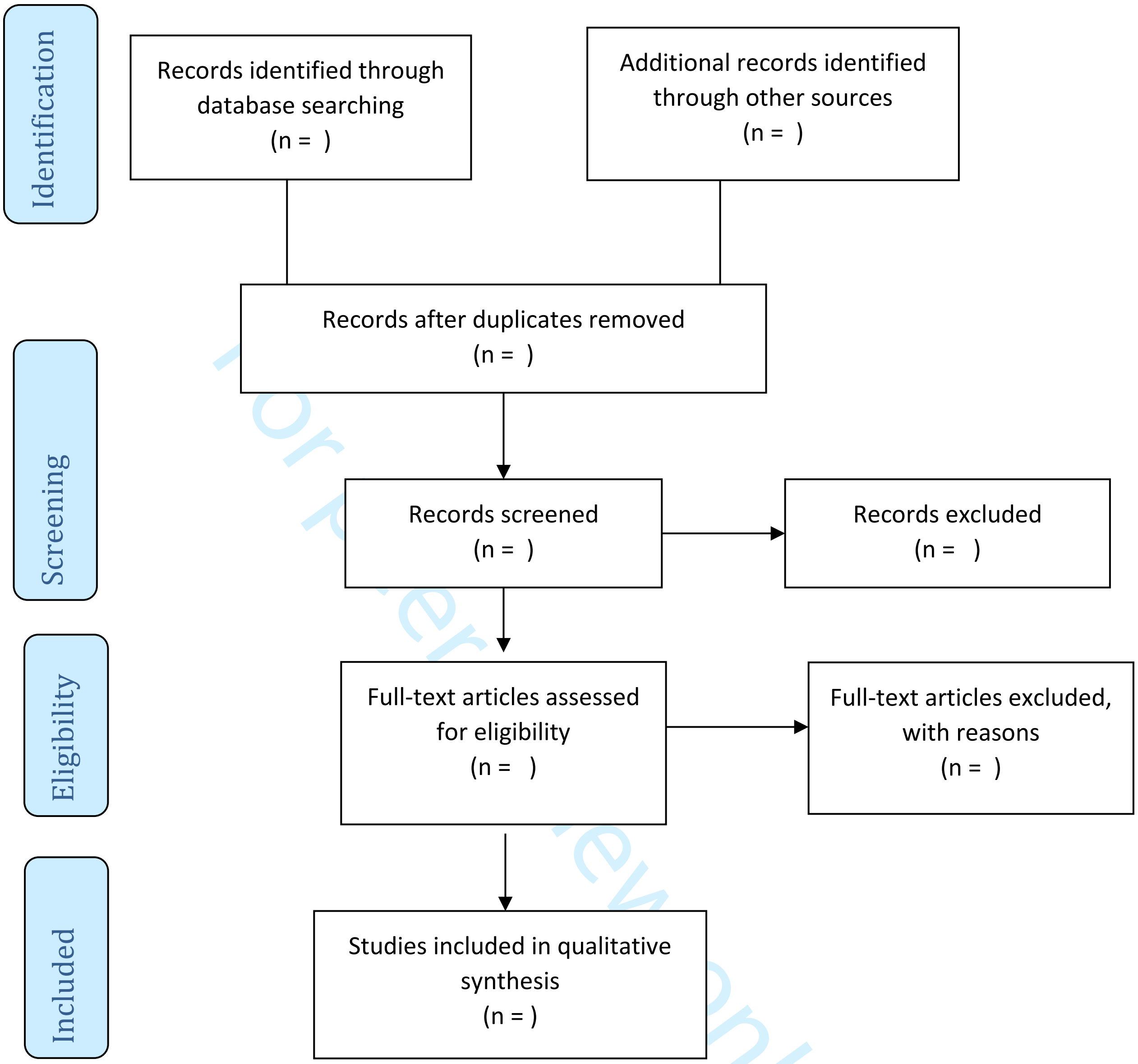

Figure 1: PRISMA 2009 Flow Diagram [19]

For peer review only - http://bmjopen.bmj.com/site/about/guidelines.xhtml

Supplementary file 1: PRISMA-P 2015 Checklist

Section/topic	#	Checklist item	Information reported Yes	Information reported No	Line number(s)
**ADMINISTRATIVE INFORMATION**					
**Title:** Mapping Evidence of Food Safety at Transport stations in Africa: A Scoping Review Protocol					
Identification	1a	Identify the report as a protocol of a systematic review	☒	☐	2
Update	1b	If the protocol is for an update of a previous systematic review, identify as such	☐	☒	
**Registration**	2	If registered, provide the name of the registry (e.g., PROSPERO) and registration number in the Abstract	☐	☒	Not applicable. This is a systematic scoping review
**Authors**					
Contact	3a	Provide name, institutional affiliation, and e-mail address of all protocol authors; provide physical mailing address of corresponding author	☒	☐	6 to 16
Contributions	3b	Describe contributions of protocol authors and identify the guarantor of the review	☒	☐	244 to 246
**Amendments**	4	If the protocol represents an amendment of a previously completed or published protocol, identify as such and list changes; otherwise, state plan for documenting important protocol amendments	☐	☒	
**Support**					
Sources	5a	Indicate sources of financial or other support for the review	☒	☐	249-251
Sponsor	5b	Provide name for the review funder and/or sponsor	☒	☐	249-251
Role of sponsor/funder	5c	Describe roles of funder(s), sponsor(s), and/or institution(s), if any, in developing the protocol	☒	☐	249-252
**INTRODUCTION**					
**Rationale**	6	Describe the rationale for the review in the context of what is already known	☒	☐	112-124
**Objectives**	7	Provide an explicit statement of the	☒	☐	124-126

Section/topic	#	Checklist item	Information reported		Line number(s)
			Yes	No	
		question(s) the review will address with reference to participants, interventions, comparators, and outcomes (PCC)			
**METHODS**					
**Eligibility criteria**	8	Specify the study characteristics (e.g., PICO, study design, setting, time frame) and report characteristics (e.g., years considered, language, publication status) to be used as criteria for eligibility for the review	☒	☐	143-146 and Table 1
**Information sources**	9	Describe all intended information sources (e.g., electronic databases, contact with study authors, trial registers, or other grey literature sources) with planned dates of coverage	☒	☐	149-150
**Search strategy**	10	Present draft of search strategy to be used for at least one electronic database, including planned limits, such that it could be repeated	☒	☐	Table 2
STUDY RECORDS					
Data management	11a	Describe the mechanism(s) that will be used to manage records and data throughout the review	☐	☐	199-201 and Figure 1
Selection process	11b	State the process that will be used for selecting studies (e.g., two independent reviewers) through each phase of the review (i.e., screening, eligibility, and inclusion in meta-analysis)	☒	☐	188-196
Data collection process	11c	Describe planned method of extracting data from reports (e.g., piloting forms, done independently, in duplicate), any processes for obtaining and confirming data from investigators	☒	☐	203-209
**Data items**	12	List and define all variables for which data will be sought (e.g., PICO items, funding sources), any pre-planned data assumptions and simplifications	☒	☐	Table 3
**Outcomes and prioritization**	13	List and define all outcomes for which data will be sought, including prioritization of	☒	☐	Table 3

For peer review only

Section/topic	#	Checklist item	Information reported		Line number(s)
			Yes	No	
		main and additional outcomes, with rationale			
**Risk of bias in individual studies**	14	Describe anticipated methods for assessing risk of bias of individual studies, including whether this will be done at the outcome or study level, or both; state how this information will be used in data synthesis	☐	☒	Not applicable
DATA: A narrative approached will be used to present results					
**Synthesis**	15a	Describe criteria under which study data will be quantitatively synthesized	☐	☒	Not applicable
	15b	If data are appropriate for quantitative synthesis, describe planned summary measures, methods of handling data, and methods of combining data from studies, including any planned exploration of consistency (e.g., I^2, Kendall's tau)	☐	☒	Not applicable
	15c	Describe any proposed additional analyses (e.g., sensitivity or subgroup analyses, meta-regression)	☐	☒	Not applicable
	15d	If quantitative synthesis is not appropriate, describe the type of summary planned	☐	☒	Not applicable
**Meta-bias(es)**	16	Specify any planned assessment of meta-bias(es) (e.g., publication bias across studies, selective reporting within studies)	☐	☒	Not applicable
**Confidence in cumulative evidence**	17	Describe how the strength of the body of evidence will be assessed (e.g., GRADE)	☐	☒	Not applicable

For peer review only

BMJ Open

Mapping Evidence of Food Safety at Transport stations in Africa: A Scoping Review Protocol

Journal:	*BMJ Open*
Manuscript ID	bmjopen-2019-035879.R2
Article Type:	Protocol
Date Submitted by the Author:	25-May-2020
Complete List of Authors:	Ncama, Busisiwe; University of KwaZulu-Natal College of Health Sciences<br>Kuupiel , Desmond ; University of KwaZulu-Natal College of Health Sciences, Public Health Medicine; Research for Sustainable Development Consult,<br>Duma, Sinegugu; University of KwaZulu-Natal College of Health Sciences<br>Mchunu , Gugu ; University of KwaZulu-Natal College of Health Sciences<br>Guga , Phindile ; University of KwaZulu-Natal College of Health Sciences<br>Slotow, Rob; University of KwaZulu-Natal
**Primary Subject Heading**:	Public health
Secondary Subject Heading:	Health policy, Public health, Evidence based practice
Keywords:	Public health < INFECTIOUS DISEASES, PREVENTIVE MEDICINE, PUBLIC HEALTH, Health policy < HEALTH SERVICES ADMINISTRATION & MANAGEMENT, Health & safety < HEALTH SERVICES ADMINISTRATION & MANAGEMENT

SCHOLARONE™
Manuscripts

I, the Submitting Author has the right to grant and does grant on behalf of all authors of the Work (as defined in the below author licence), an exclusive licence and/or a non-exclusive licence for contributions from authors who are: i) UK Crown employees; ii) where BMJ has agreed a CC-BY licence shall apply, and/or iii) in accordance with the terms applicable for US Federal Government officers or employees acting as part of their official duties; on a worldwide, perpetual, irrevocable, royalty-free basis to BMJ Publishing Group Ltd ("BMJ") its licensees and where the relevant Journal is co-owned by BMJ to the co-owners of the Journal, to publish the Work in this journal and any other BMJ products and to exploit all rights, as set out in our licence.

The Submitting Author accepts and understands that any supply made under these terms is made by BMJ to the Submitting Author unless you are acting as an employee on behalf of your employer or a postgraduate student of an affiliated institution which is paying any applicable article publishing charge ("APC") for Open Access articles. Where the Submitting Author wishes to make the Work available on an Open Access basis (and intends to pay the relevant APC), the terms of reuse of such Open Access shall be governed by a Creative Commons licence – details of these licences and which Creative Commons licence will apply to this Work are set out in our licence referred to above.

Other than as permitted in any relevant BMJ Author's Self Archiving Policies, I confirm this Work has not been accepted for publication elsewhere, is not being considered for publication elsewhere and does not duplicate material already published. I confirm all authors consent to publication of this Work and authorise the granting of this licence.

For peer review only

Mapping Evidence of Food Safety at Transport stations in Africa: A Scoping Review Protocol

Busisiwe P. Ncama[1], Desmond Kuupiel[1,2], Sinegugu E. Duma[1], Gugu Mchunu[1], Phindile Guga[1], Rob Slotow[3]

[1]School of Nursing and Public Health, College of Health Sciences, University of KwaZulu-Natal, Durban, South Africa

[2]Research for Sustainable Development Consult, Sunyani, Ghana

[3]School of Life Sciences, College of Agriculture, Engineering and Science, University of Kwazulu-Natal, Pietermaritzburg, South Africa.

Corresponding Author: Desmond Kuupiel (Ph.D.)

Address: 2nd Floor George Campbell Building, Department of Public Health Medicine, School of Nursing and Public Health, University of KwaZulu-Natal, Durban, 4001, South Africa

Email address: desmondkuupiel98@hotmail.com / KuupielD@ukzn.ac.za

Tel: +27735568200/ +233550972968

Email addresses of co-authors

Prof. Busisiwe P. Ncama (Ncamab@ukzn.ac.za)

Prof. Sinegugu E. Duma (DumaS1@ukzn.ac.za)

Dr. Gugu Mchunu (Mchunug@ukzn.ac.za)

Ms. Phindile Guga (phindile.guga@gmail.com)

Prof. Rob Slotow (Slotow@ukzn.ac.za)

For peer review only - http://bmjopen.bmj.com/site/about/guidelines.xhtml

Abstract

Introduction: In Africa, travels, urbanization, and changing consumer habits, are increasing the number of people buying and eating food prepared/sold at public spaces including transport stations, particularly in the urban and peri-urban areas. Although food trading in such public spaces serves as a source of livelihood for many people, unsafe food can have a negative impact on health. We, therefore, aim to systematically explore and examine the literature, and describe the evidence on food safety (food handling, storage, preparation and sale, packaging of food when sold, hygiene of sale venue, and quality (nutrition) of food sold/purchased/eaten) at transport stations to inform policy, as well as identify research gaps for future studies in Africa.

Methods and Analysis: We will employ the Arksey & O'Malley framework, Levac et al. recommendations, and the Joanna Briggs Institute guidelines to guide this study. We will conduct a comprehensive search in PubMed, SCOPUS, Web of Science, Google Scholar, and EBSCOhost (Academic search complete, CINAHL with Full-text, and Health Source) from inception to December 2019 for relevant peer-review articles using a combination of keywords/search terms with no limitations. We will also search for relevant literature from the reference list of all included articles. Two investigators will independently screen the articles in parallel at the abstract and full-text phases using the eligibility criteria as a guide. Data extraction will be done using a piloted data extraction form designed in a Microsoft ® Word tabular format. Afterward, the extracted data will be collated into themes and sub-themes, summarized, and the results reported utilizing a narrative approach. We will the Preferred Reporting Items for Systematic Reviews and Meta-analyses: Extension for scoping reviews checklist to report this study results.

Ethics and Dissemination: Ethics approval not required. All sources of data will be adequately cited and added to the reference list. We will present the final scoping review results at the appropriate workshops, meetings, conferences, as well as submit for peer review and publication in a scientific journal.

Keywords: Food safety, Transport stations, Taxi ranks, Car parks, Bus stops, Automobile stations, Africa

For peer review only

Article summary

Strengths and limitations of this study

- This will be the first scoping review to systematically explore literature and describe the evidence on food safety at transport stations to inform policy, as well as identify research gaps for future studies in Africa
- A comprehensive search for potentially eligible articles will be conducted in four electronic databases, and websites of international organizations.
- The scoping review permits the inclusion of all types of study designs, and it encompasses both published and grey literature.
- Boolean words AND/OR as well as medical subject heading (MeSH) terms will be included in the electronic search strategy to capture all relevant studies.
- This study limitation is the use of only four databases, which may potentially result in missing relevant articles indexed in other electronic databases.

For peer review only - http://bmjopen.bmj.com/site/about/guidelines.xhtml

Background

Food is one of the major modes of disease transmission for microbial, chemical, and physical hazards. Food containing harmful bacteria, viruses, parasites or chemical substances can cause more than 200 different diseases – ranging from diarrhea to cancers [1]. Although unsafe food can have a significant impact on health in both high-income and low-and middle-income countries (LMICs), the impact is much higher in LMICs [2, 3]. Globally, it is estimated that unsafe food results in about 600 million cases of foodborne diseases and close to 420 000 deaths with about 30% of all foodborne deaths occurring among children less than 5 years annually [1, 2]. The WHO further estimates that eating unsafe food results in approximately 33 million years of healthy lives lost (DALYs) worldwide every year [1]. As a result, in 2006, the World Health Organization (WHO) declared foodborne diseases as well as food-related diseases and food safety as a major public health concern [4, 5]. Unsafe food creates a vicious cycle of disease and malnutrition, particularly affecting infants, young children, the elderly, and the sick [6]. To this end, access to safe, and nutritious and sufficient food is key to sustaining life and promoting good health.

Food safety may denote the prevention of biological or non-biological hazards in food, whether chronic or acute, that may make food harmful to the health of the consumer [7]. Food safety is about producing, handling, storing and preparing food in such a way as to prevent infection and contamination in the food production chain, and to help ensure that food quality and wholesomeness are maintained to promote good health [7]. In addition to contributing to food and nutrition security, a safe food supply also supports national economies, trade, and tourism, stimulating sustainable development [7, 8]. The globalization of food trade, a growing world population, climate change and rapidly changing food systems have an impact on the safety of food [7, 8].

In most LMICs, food trading at transport stations such as taxi ranks, bus stations, and lorry parks is fast-growing, particularly in the urban and peri-urban areas. It is evident that over 2.4 billion people eat food sold by vendors at public spaces including transport stations every day worldwide [9, 10]. This may be due to several reasons such as the thinking that the foods sold in public spaces are appealing, ready-to-eat, convenience, and of low cost and savory taste [11-13]. Food trading in public spaces serves as a source of livelihood for many households in LMICs [8, 11, 13]. Although the food sold at transport stations may also have great nutritional value depending on the type of ingredients and the conditions under which the food was prepared and served to the customer, it may as well cause ill-health arising from contamination

For peer review only - http://bmjopen.bmj.com/site/about/guidelines.xhtml

of the food. Since the food is most often prepared and sold in an environment that often lacks sanitary facilities and health surveillance, and at times, not regulated by the appropriate local authorities in most LMICs [13-15]. Despite this, no study to the best of our knowledge has mapped literature on food safety at transport stations in Africa. We, therefore, intend to systematically search for literature, examine the scope of the literature and describe the evidence on food safety at transport stations in Africa in order to inform policy. We also anticipate identifying gaps from the study findings for future research to improve food safety at transport stations such as taxi ranks, bus stations, and lorry parks in Africa.

Methods

Design of the study protocol

We will employ an amended Arksey & O'Malley framework incorporating the Levac et. al. recommendation [16, 17] to guide this scoping review. The Arksey & O'Malley framework includes the following: research question identification; identifying relevant studies; selection of study; data charting, collating, summarising and reporting the findings, and consultation. However, we will exclude the consultation stage because this review is nested in a larger study that already includes stakeholder consultation. We followed the preferred reporting items for systematic and meta-analysis extension for protocols (PRISMA-P) guidelines to develop this protocol (Supplementary file 1). However, we will the Preferred Reporting Items for Systematic Reviews and Meta-analyses: Extension for scoping reviews checklist to report this study results.

Identifying the research question

This scoping review question shall be:

What is the scope of evidence on food safety at transport stations in Africa? Table 1 presents the Population, Concept, and Context (PCC) mnemonic used to inform the eligibility of this proposed review [18].

Identify relevant studies

We will conduct a comprehensive search in PubMed, SCOPUS, Google scholar, Web of Science, and EBSCOhost (Academic search complete, CINAHL with Full-text, and Health Source) from inception to December 2019 for relevant articles. We will develop the search strategy in consultation with a subject Librarian at the University of KwaZulu-Natal so as to enable us capture all/most relevant articles for the study. We will use a combination of the

For peer review only - http://bmjopen.bmj.com/site/about/guidelines.xhtml

following keywords: “transport station” OR “taxi rank” OR “automobile station” OR “bus station” OR “lorry park” OR “car park” OR “transport hub” OR “streets” OR “public space” AND "food safety" OR "food handling" OR "food storage" OR “food preparation” OR “food packaging” OR “food sale” OR “food vend” OR “food sale” OR “food sold” OR “food hawking” OR “food purchase” in order to enable us capture all appropriate peer-review articles. We will include Booleans terms AND/OR and medical subject heading (MeSH) terms in our search. Date, study design, and language limitations will be removed during the databases search. We will also search the reference list of all included studies for articles presenting useful information to address this proposed scoping review question. The search date, database, keywords, number of retrieved articles, number of eligible articles, and all other relevant search records will be documented appropriately in detail. Table 2 illustrates a full search strategy in PubMed database for this proposed scoping review study.

Eligibility criteria and study selection

The eligibility criteria will be as outlined below.

Inclusion criteria:

This shall include the following:

- Studies reporting evidence from Africa,
- Studies presenting evidence on food safety,
- Studies reporting from transport stations,
- Studies published in English, and
- Primary studies focusing on food safety at transportations

Exclusion criteria

We shall exclude the following:

- Studies presenting evidence from other countries outside Africa,
- Studies presenting evidence of food safety from other sites such as homes, restaurants, markets, and hotels, and schools,
- Studies published in other languages such as French, Arabic, and Portuguese, and
- Review studies

Study selection

The screening for potentially eligible studies will be done in three phases. In phase one, a

peer review only

For peer review only - http://bmjopen.bmj.com/site/about/guidelines.xhtml

comprehensive article search in the electronic databases will be conducted by one investigator and import all eligible articles to a new endnotes X9 library created for the review. To clean the library, all duplicates will be deleted and subsequently shared with the co-investigators. In phase two, two investigators will independently screen the abstracts in parallel guided by the eligibility criteria designed using a google form. All disagreements between the investigators in terms of their response at this stage will be addressed through discussions among the investigators until a consensus is reached. Similarly, in the third phase, two investigators will independently screen all the full-text articles as described in phase two, however, a third investigator will be engaged to resolve any discrepancies in their responses. Cohen's kappa statistic will be calculated following full text screening to demonstrate the level of agreement between reviewers. All articles retrieved from the reference list of included studies will also be screened using the same process. Where a full-text article cannot be retrieved or not accessible from the online databases, we will seek assistance from the University of KwaZulu-Natal library or write to the authors to request the full-text for screening. An amended PRISMA (Preferred Reporting Items for Systematic Review and Meta-Analyses) flow diagram will be used to report the search and screening results [19] (Figure 1).

Charting the data

We will design a data extraction form using Microsoft ® Word in a tabular format. To ensure the trustworthiness and reliability of the data, two independent investigators will pilot the data extraction form using a random sample of ten percent of the included articles in parallel. Subsequently, the data extraction form will be revised if necessary, to ensure its rigor and ability to capture all relevant data to answer the review question. We will keep the data extraction form updated throughout this process until all relevant information has been extracted from the included articles. Table 3 shows the data extraction form that will be used for this scoping review study.

Collating, summarizing, and reporting the results

We will present information about the included articles that are aligned with the research question of this proposed scoping review study. All relevant data from the eligible studies will be extracted using the piloted data-extraction form to answer the review question. Both deductive (for characteristics of the included articles) and inductive (for study findings/outcomes) approaches will be used to extract relevant data. Thematic analysis will be

For peer review only - http://bmjopen.bmj.com/site/about/guidelines.xhtml

utilised to collate the study findings into themes and sub-themes. Then, a narrative summary of the themes and sub-themes will be reported. To ensure rigor, the analysis will be conducted by two investigators with input from collaborators. The PRISMA extension designed for the scoping reviews checklist will be utilized to guide the reporting of the results of this scoping review. Afterward, a meta-analysis may be conducted using quantitative data if possible.

Ethics and dissemination

Not required. All sources of data will be adequately referenced. We will present the final scoping review results at the appropriate workshops, meetings, conferences, as well as submit for peer review and publication in a scientific journal.

Patient and Public Involvement

No patient involved

Statements

Acknowledgments

Support for this work was provided by the Sustainable and Healthy Food Systems (SHEFS) Programme, supported through the Wellcome Trust's Our Planet, Our Health Programme

Authors' contributions

BPN, DK, SED, SM, and RS conceptualized the study. DK wrote the manuscript and PG contributed to the writing. BPN, SED, GM, and RS critically review the manuscript and made revisions. DK wrote the final draft manuscript and all the authors approved it.

Funding

Funding for this work was provided by the Sustainable and Healthy Food Systems (SHEFS) Programme, supported through the Wellcome Trust's Our Planet, Our Health Programme [grant number: 205200/Z/16/Z]. The funder played no role in the literature search and writing of the manuscript.

Competing interests

None declared

References

For peer review only

For peer review only - http://bmjopen.bmj.com/site/about/guidelines.xhtml

1. World Health Organization. Estimating the burden of foodborne diseases Geneva: World Health Organization; 2015 [cited 2019 25/10/2019]. Available from: https://www.who.int/activities/estimating-the-burden-of-foodborne-diseases.

2. The Lancet Gastroenterology H. Food safety really is everyone's business. The lancet Gastroenterology & hepatology. 2019;4(8):571.

3. Allard DG. The 'farm to plate' approach to food safety - Everyone's business. The Canadian journal of infectious diseases = Journal canadien des maladies infectieuses. 2002;13(3):185-90.

4. World Health Organization. Five keys to safer food manual Geneva: World Health Organization; 2006 [cited 2019 25/10/2019]. Available from: https://www.who.int/foodsafety/publications/consumer/manual_keys.pdf.

5. Lamin-Boima P. Knowledge, Attitude And Practice Of Street Food Vendors In Selected Schools Within Bo City Southern Sierra Leone.

6. World Health Organization. Food safety Geneva: World Health Organization; 2019 [cited 2020 25/03/2020]. Available from: https://www.who.int/news-room/fact-sheets/detail/food-safety.

7. Organization WH. Food Safety: What you should know Geneva: World Health Organization; 2015 [cited 2019 25/10/2019]. Available from: http://www.searo.who.int/entity/world_health_day/2015/whd-what-you-should-know/en/.

8. Hill J, McHiza Z, Puoane T, Steyn NP. The development of an evidence-based street food vending model within a socioecological framework: A guide for African countries. PloS one. 2019;14(10):e0223535.

9. Food and Agriculture Organization. Selling street and snack foods Rome: Food and Agriculture Organization; 2011 [cited 2019 25/10/2019]. Available from: http://www.fao.org/3/a-i2474e.pdf.

10. Gelormini M, Damasceno A, Lopes SA, Malo S, Chongole C, Muholove P, et al. Street Food Environment in Maputo (STOOD Map): a Cross-Sectional Study in Mozambique. JMIR research protocols. 2015;4(3):e98.

11. Liu Z, Zhang G, Zhang X. Urban street foods in Shijiazhuang city, China: Current status, safety practices and risk mitigating strategies. Food Control. 2014;41:212-8.

For peer review only - http://bmjopen.bmj.com/site/about/guidelines.xhtml

12. Al Mamun M, Turin TC. Safety of Street foods. Food Hygiene and Toxicology in Ready-to-Eat Foods: Elsevier; 2016. p. 15-29.

13. Abrahale K, Sousa S, Albuquerque G, Padrao P, Lunet N. Street food research worldwide: a scoping review. Journal of human nutrition and dietetics : the official journal of the British Dietetic Association. 2019;32(2):152-74.

14. Ekanem EO. The street food trade in Africa: safety and socio-environmental issues. Food Control. 1998;9(4):211-5.

15. Rane S. Street vended food in developing world: hazard analyses. Indian journal of microbiology. 2011;51(1):100-6.

16. Arksey H, O'Malley L. Scoping studies: towards a methodological framework. International Journal of Social Research Methodology. 2005;8(1):19-32.

17. Levac D, Colquhoun H, O'Brien KK. Scoping studies: advancing the methodology. Implementation Science. 2010;5(1):69.

18. Institute JB. Joanna Briggs Institute reviewers' manual: 2015 edition/supplement. Methodology for JBI Scoping Reviews Adelaide: The Joanna Briggs Institute. 2015.

19. Moher D, Liberati A, Tetzlaff J, Altman DG, The PRISMA Group. PRISMA 2009 Flow Diagram. 2009;6(2009):1000097.

Tables

For peer review only - http://bmjopen.bmj.com/site/about/guidelines.xhtml

Table 1: PCC framework for defining the eligibility of the studies for the primary research question

P- Population	Transport stations: This will include bus stations, lorry parks, taxi ranks, and automobile stations, and bus stops.
C-Concept	Food safety: This include food handling, storage, preparation and sale, packaging of food when sold, hygiene of sale venue, and quality (nutrition) of food sold/purchased/eaten.
C-Context	African

For peer review only - http://bmjopen.bmj.com/site/about/guidelines.xhtml

Table 2: A full search strategy in PubMed illustrating the feasibility of the proposed scoping review

Date	Database	Keywords	Search results
06/04/2020	PubMed	(("transport"[All Fields]) AND station[All Fields]) OR (taxi[All Fields] AND rank[All Fields]) OR (("automobiles"[MeSH Terms] OR "automobiles"[All Fields] OR "automobile"[All Fields]) AND station[All Fields]) OR "bus station"[All Fields] OR (("motor vehicles"[MeSH Terms] OR ("motor"[All Fields] AND "vehicles"[All Fields]) OR "motor vehicles"[All Fields] OR "lorry"[All Fields]) AND park[All Fields]) OR "car park"[All Fields] OR "transport hub"[All Fields] OR "streets"[All Fields] OR "public space"[All Fields] AND "food safety"[All Fields] OR "food handling"[All Fields] OR "food storage"[All Fields] OR "food preparation"[All Fields] OR "food packaging"[All Fields] OR "food sale"[All Fields] OR (("food"[MeSH Terms] OR "food"[All Fields]) AND vend[All Fields]) OR "food sale"[All Fields] OR "food sold"[All Fields] OR (("food"[MeSH Terms] OR "food"[All Fields]) AND ("hawks"[MeSH Terms] OR "hawks"[All Fields] OR "hawking"[All Fields])) OR "food purchase"[All Fields] AND (("0000/01/01"[PDAT] : "2019/12/31"[PDAT]) AND "humans"[MeSH Terms])	13644

For peer review only

For peer review only - http://bmjopen.bmj.com/site/about/guidelines.xhtml

Table 3: Data Extraction Form

Author and publication year
Study Title
Study aim/objective
Type of study design
Study setting (Country)
Study participants
Significant findings
Food safety outcomes
Conclusions/recommendations

Figure

Figure 1: PRISMA 2009 Flow Diagram

Supplementary File

Supplementary file 1: PRISMA-P Checklist

For peer review only - http://bmjopen.bmj.com/site/about/guidelines.xhtml

Figure

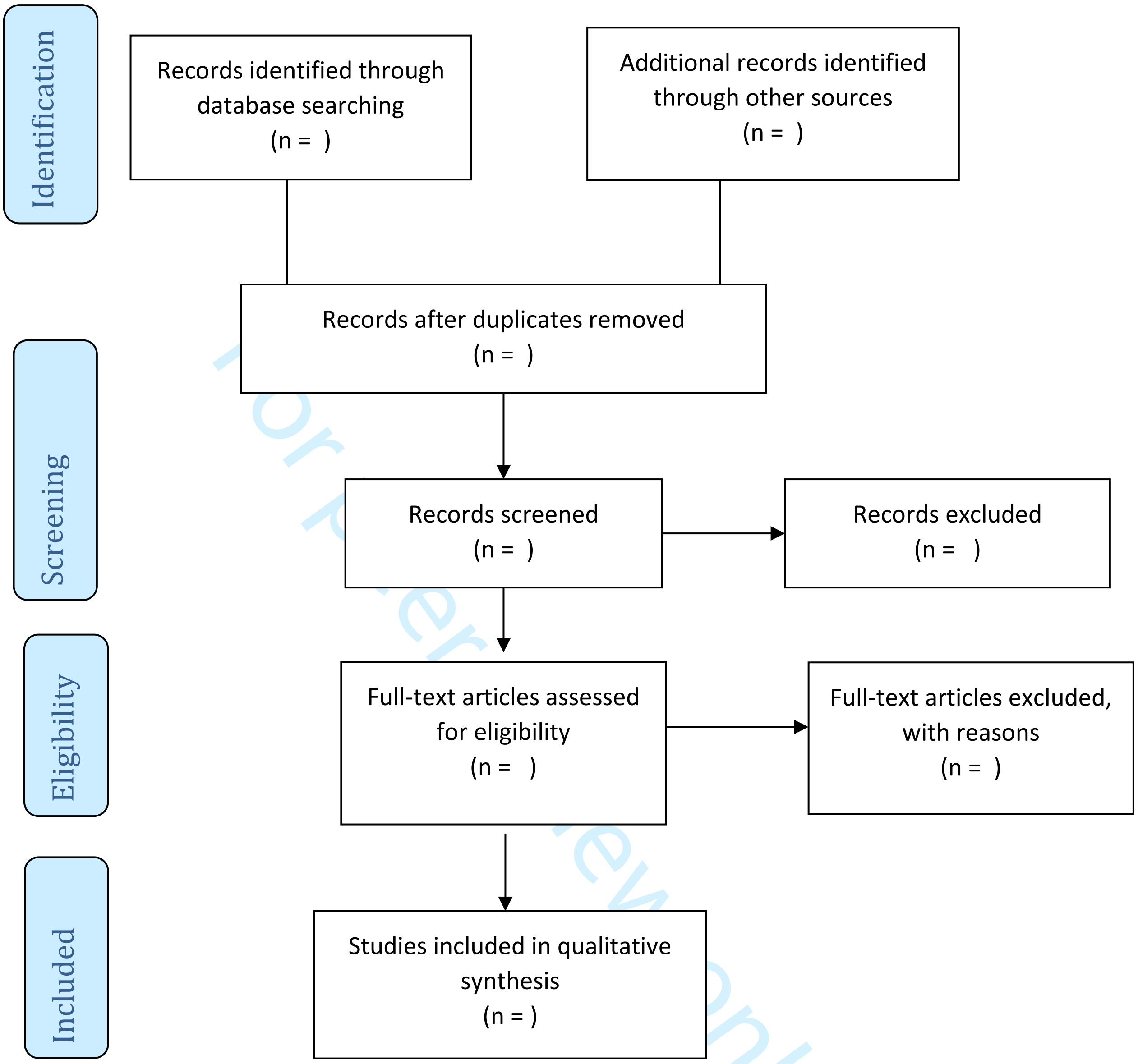

Figure 1: PRISMA 2009 Flow Diagram [19]

For peer review only - http://bmjopen.bmj.com/site/about/guidelines.xhtml

Supplementary file 1: PRISMA-P 2015 Checklist

Section/topic	#	Checklist item	Information reported		Line number(s)
			Yes	No	
**ADMINISTRATIVE INFORMATION**					
**Title:** Mapping Evidence of Food Safety at Transport stations in Africa: A Scoping Review Protocol					
Identification	1a	Identify the report as a protocol of a systematic review	☒	☐	2
Update	1b	If the protocol is for an update of a previous systematic review, identify as such	☐	☒	
**Registration**	2	If registered, provide the name of the registry (e.g., PROSPERO) and registration number in the Abstract	☐	☒	Not applicable. This is a systematic scoping review
**Authors**					
Contact	3a	Provide name, institutional affiliation, and e-mail address of all protocol authors; provide physical mailing address of corresponding author	☒	☐	6 to 16
Contributions	3b	Describe contributions of protocol authors and identify the guarantor of the review	☒	☐	244 to 246
**Amendments**	4	If the protocol represents an amendment of a previously completed or published protocol, identify as such and list changes; otherwise, state plan for documenting important protocol amendments	☐	☒	
**Support**					
Sources	5a	Indicate sources of financial or other support for the review	☒	☐	249-251
Sponsor	5b	Provide name for the review funder and/or sponsor	☒	☐	249-251
Role of sponsor/funder	5c	Describe roles of funder(s), sponsor(s), and/or institution(s), if any, in developing the protocol	☒	☐	249-252
**INTRODUCTION**					

For peer review only

Section/topic	#	Checklist item	Information reported		Line number(s)
			Yes	No	
**Rationale**	6	Describe the rationale for the review in the context of what is already known	☒	☐	112-124
**Objectives**	7	Provide an explicit statement of the question(s) the review will address with reference to participants, interventions, comparators, and outcomes (PCC)	☒	☐	124-126
**METHODS**					
**Eligibility criteria**	8	Specify the study characteristics (e.g., PICO, study design, setting, time frame) and report characteristics (e.g., years considered, language, publication status) to be used as criteria for eligibility for the review	☒	☐	143-146 and Table 1
**Information sources**	9	Describe all intended information sources (e.g., electronic databases, contact with study authors, trial registers, or other grey literature sources) with planned dates of coverage	☒	☐	149-150
**Search strategy**	10	Present draft of search strategy to be used for at least one electronic database, including planned limits, such that it could be repeated	☒	☐	Table 2
STUDY RECORDS					
Data management	11a	Describe the mechanism(s) that will be used to manage records and data throughout the review	☐	☐	199-201 and Figure 1
Selection process	11b	State the process that will be used for selecting studies (e.g., two independent reviewers) through each phase of the review (i.e., screening, eligibility, and inclusion in meta-analysis)	☒	☐	188-196
Data collection process	11c	Describe planned method of extracting data from reports (e.g., piloting forms, done independently, in duplicate), any processes for obtaining and confirming data from investigators	☒	☐	203-209
**Data items**	12	List and define all variables for which data	☒	☐	Table 3

For peer review only

Section/topic	#	Checklist item	Information reported: Yes	Information reported: No	Line number(s)
		will be sought (e.g., PICO items, funding sources), any pre-planned data assumptions and simplifications			
**Outcomes and prioritization**	13	List and define all outcomes for which data will be sought, including prioritization of main and additional outcomes, with rationale	☒	☐	Table 3
**Risk of bias in individual studies**	14	Describe anticipated methods for assessing risk of bias of individual studies, including whether this will be done at the outcome or study level, or both; state how this information will be used in data synthesis	☐	☒	Not applicable
DATA: A narrative approached will be used to present results					
**Synthesis**	15a	Describe criteria under which study data will be quantitatively synthesized	☐	☒	Not applicable
	15b	If data are appropriate for quantitative synthesis, describe planned summary measures, methods of handling data, and methods of combining data from studies, including any planned exploration of consistency (e.g., I^2, Kendall's tau)	☐	☒	Not applicable
	15c	Describe any proposed additional analyses (e.g., sensitivity or subgroup analyses, meta-regression)	☐	☒	Not applicable
	15d	If quantitative synthesis is not appropriate, describe the type of summary planned	☐	☒	Not applicable
**Meta-bias(es)**	16	Specify any planned assessment of meta-bias(es) (e.g., publication bias across studies, selective reporting within studies)	☐	☒	Not applicable
**Confidence in cumulative evidence**	17	Describe how the strength of the body of evidence will be assessed (e.g., GRADE)	☐	☒	Not applicable

For peer review only
